# Impaired booster-induced SARS-CoV-2 antibody responses in rituximab-treated B-cell lymphoma patients despite peripheral B-Cell Recovery

**DOI:** 10.2478/raon-2026-0014

**Published:** 2026-03-24

**Authors:** Tomaz Jurca, Lucka Boltezar, Miha Orazem, Kristina Fujs Komlos, Katka Pohar, Sara Jevnikar, Mario Poljak, Denis Mlakar Mastnak, Nada Rotovnik Kozjek, Bor Vratanar, Janja Ocvirk, Alojz Ihan

**Affiliations:** Institute of Oncology Ljubljana, Ljubljana, Slovenia; Faculty of Medicine, University of Ljubljana, Ljubljana, Slovenia; Institute of Microbiology and Immunology, Faculty of Medicine, University of Ljubljana, Ljubljana, Slovenia; Institute for Biostatistics and Medical Informatics, Faculty of Medicine, University of Ljubljana, Ljubljana, Slovenia; Faculty of Health Sciences, University of Primorska, Izola, Slovenia

**Keywords:** lymphoma, B-cell, rituximab, COVID-19 vaccines, humoral immunity, antibodies, viral

## Abstract

**Background:**

Rituximab-treated patients with B-cell lymphoma exhibit profound B-cell depletion and impaired vaccine-induced antibody responses. However, it remains uncertain whether recovery of peripheral B-cell counts after rituximab is sufficient to restore effective humoral immunity following booster vaccination.

**Patients and methods:**

In this prospective, single-center observational study at the Institute of Oncology Ljubljana, adult B-cell lymphoma patients treated with or previously exposed to rituximab received the Comirnaty® mRNA COVID-19 vaccine. Antibody responses to the SARS-CoV-2 spike and nucleoprotein were measured at baseline, 14 days after the second dose, and at 3, 6, 9, and 12 months. A third dose was administered at 6 months. T-cell responses (IFN-γ release) were assessed in patients before and after the primary series and booster dose. Lymphocyte subsets were analysed pre-vaccination. Adverse events and nutritional status were monitored.

**Results:**

Patients undergoing anti-CD20 therapy showed absent antibody responses. Longer intervals since rituximab correlated with peripheral B-cell repopulation. However, even after reaching normal B-cell counts, patients’ antibody responses after revaccination remained significantly lower than in controls.

**Conclusions:**

Rituximab is associated with impaired vaccine-induced antibody responses. Despite recovery of peripheral B-cell counts, patients with B-cell lymphoma show reduced humoral responses compared with healthy individuals following booster COVID-19 vaccination.

## Introduction

Cancer patients are significantly more vulnerable to COVID-19 infection due to their immunocompromised status, resulting from both the malignancy itself and the effects of oncological treatments.^1,2^ Consequently, this population is at increased risk of severe disease and higher mortality compared with the general population.^2-4^ Vaccination against COVID-19 is therefore crucial for protecting cancer patients; however, the effectiveness of vaccination is reduced by certain anticancer therapies.^5-7^

In particular, anti-CD20 monoclonal antibody therapy, commonly used in the treatment of B-cell lymphomas, induces prolonged B-cell depletion, leading to impaired antibody production and compromised humoral immunity.^8,9^ As a result, vaccine-induced antibody responses, including those elicited by COVID-19 vaccines and other routinely administered immunizations, are substantially diminished in a significant proportion of these patients.^10,11^

The association between anti-CD20 therapy and reduced vaccine responsiveness is well established, and higher peripheral B-lymphocyte counts have been associated with improved antibody responses following vaccination in patients with haematological malignancies^10-12^ and autoimmune diseases.^13,14^

However, it remains uncertain whether recovery of peripheral B-cell counts after rituximab is sufficient to restore effective humoral immunity following booster vaccination.^11,15-18^

This prospective, non-interventional clinical study aimed to investigate the immune response elicited by COVID-19 vaccination in patients with B-cell lymphoma undergoing different treatment modalities.

We hypothesized that B-cell lymphoma patients receiving anti-CD20 therapy would show no antibody response to vaccination, with increasingly better responses as more time elapsed since their last rituximab treatment. A stronger response was expected in patients who had previously been infected with COVID-19 and subsequently received vaccination. We also anticipated more pronounced vaccine-related side effects in those with a stronger immune response. We anticipated preserved T-cell response, except in patients receiving concurrent chemotherapy.

## Patients and methods

### Study design

Adult patients with B-cell lymphoma were prospectively enrolled in a single-center observational study at the Institute of Oncology Ljubljana, Slovenia. The control group consisted of healthcare employers. Once the vaccine became available and the protocol was approved by the ethics committee, we conducted telephone interviews with all patients who met the inclusion criteria. Approximately one third of eligible patients were included. Another third had already been vaccinated, and the remaining third declined vaccination. Patient recruitment took place from early April to the end of May 2021.

Vaccination was performed using the Comirnaty® mRNA vaccine according to the manufacturer’s (Pfizer-BioNTech) guidelines. Participation in the study did not influence the course of lymphoma treatment.

Prior to vaccination, lymphocyte populations were analysed in patients. The B-cell and T-cell responses to the viral nucleoprotein (N) and spike (S) protein were assessed before the first vaccination and again 14 days after the second and third dose. Anti-N and anti-S antibody concentrations were monitored every three months for one year to evaluate the increase and decline of post-vaccination antibodies (anti-S) and to detect potential infections through the appearance of anti-N antibodies.

**FIGURE 1.**
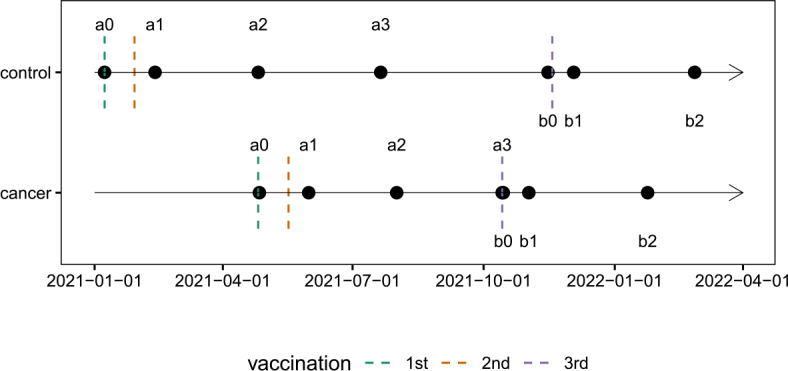
The timeline of vaccinations and laboratory analyses. Time points of sample collection are indicated as follows: a0 – baseline measurement and first vaccination (second dose administered 21 days later); a1 – 14 days after the second vaccination; a2 – 3 months after the first vaccination; a3 – 6 months after the first vaccination (corresponding to b0 in cancer patients); b0 – before the third vaccination; b1 – 14 days after the third vaccination; b2 – 3 months after the third vaccination.

Patients received the 3rd dose at their 6-month follow-up visit, while volunteers who had been vaccinated earlier, received the 3rd dose 9 months after the first dose. The timeline of vaccinations and laboratory analyses is presented in Figure 1.

### Inclusion criteria

We included patients with a histologically confirmed diagnosis of B-cell lymphoma who agreed to participate in the study. Eligible patients had to have received rituximab at some point during their disease course and be between 18 and 80 years of age.

### Exclusion criteria

Patients with a known immunodeficiency (e.g., HIV infection, history of solid organ transplantation) and those receiving immunosuppressive therapy (e.g., long-term glucocorticoid treatment, interleukin therapy, TNF-alpha inhibitors) were excluded from the study. We also excluded patients who had not yet received treatment for their lymphoma and those who had received intravenous immunoglobulin within the previous six months.

### Patient population

The study population consisted of patients with B-cell lymphoma across various histological subtypes:

Group L1 included patients with high-grade B-cell lymphoma treated with rituximab and the regimen with cyclophosphamide, doxorubicin, vincristine, and prednisolone (CHOP) who received the vaccine during systemic treatment. Group L2 included patients with follicular or mantle cell lymphoma receiving rituximab maintenance therapy. Group L3 included patients with various B-cell lymphoma subtypes who had completed rituximab plus chemotherapy treatment (primarily rituximab -CHOP [R-CHOP] or R-CHOP-like regimens) within 24 months before receiving the first vaccine dose. Group L4 included patients with various B-cell lymphoma subtypes treated with rituximab, either as monotherapy or in combination with chemotherapy, more than 24 months before receiving their first SARS-CoV-2 vaccine dose.

Sample size estimation was based on the first published data. To detect a 50% reduction in B cell response, 160 participants (4 groups of 40) were calculated to be required. Healthy volunteers served as the control group.

However, due to the very low number of enrolled patients, the originally planned group structure was not feasible, and the primary objective was modified to enable a meaningful exploratory analysis.

We combined participants in groups L1 and L2 into the “on-rituximab” group and those in groups L3 and L4 into the “post-rituximab” group. Analysis was only possible in the latter group. Post-rituximab patients were further stratified by B-lymphocyte count, measured before the 1st vaccination, into a normal B-lymphocyte group (B > 0.1 × 10^9^/L), a reduced B-lymphocyte group (B < 0.1 × 10^9^/L), or an absent B-lymphocyte group.

Due to the small sample size, we excluded individuals who contracted COVID-19. We also excluded participants with missing anti-S values, those lacking B-lymphocyte count data, and one individual whose interval since the last rituximab dose was exceptionally long (10 years).

Patients and controls were followed for one year. Because the control group did not have a sixmonth post-revaccination assessment due to revaccination at nine months, we also excluded the b3 measurement (six months after revaccination) from the patients’ analysis.

### Variables

Primary outcomes were anti-spike (anti-S) antibody concentration and interferon-gamma (IFN-γ) T-cell responses following primary and booster vaccinations. Secondary outcomes included vaccine-related adverse events and key nutritional and physical-functional parameters.

The main explanatory variable was the prevaccination B-lymphocyte count; patients were stratified into three groups based on whether their B-cell counts were within the normal range, reduced or absent.

Potential confounders comprised age, sex and comorbidities.

### Methods

#### Enumeration of lymphocyte subsets using flow cytometry

For enumeration of peripheral lymphocyte subsets, 50 μL of fresh peripheral blood was stained using the BD Multitest™ 6-Color TBNK reagent (Becton Dickinson, Franklin Lakes, New Jersey, USA), containing monoclonal antibodies CD3 FITC, CD16+CD56 PE, CD45 PerCP-Cy5.5, CD4 PE-Cy7, CD19 APC, and CD8 APC-Cy7, together with BD Trucount™ beads (Becton Dickinson, Franklin Lakes, New Jersey, USA). After a 20-minute incubation, red blood cells were lysed and samples were fixed by adding 450 μL of BD FACS Lysing Solution™ (Becton Dickinson, Franklin Lakes, New Jersey, USA) followed by a 15-minute incubation. Samples were acquired within 1 hour on a BD FACSCanto II flow cytometer and analysed using BD FACSCanto™ Clinical Software (Becton Dickinson, Franklin Lakes, New Jersey, USA).

#### B-cell antibody response

It was measured in plasma using the Elecsys Anti-SARS-CoV-2 S test (Roche Diagnostics, Mannheim, Germany) for the quantitative detection of total anti-SARS-CoV-2 antibodies against the S protein receptor-binding domain (RBD), and the Elecsys Anti-SARS-CoV-2 test (Roche Diagnostics, Mannheim, Germany) for the qualitative detection of total anti-SARS-CoV-2 antibodies against the N protein. Testing was performed on the cobas e411 analyzer (Roche Diagnostics, Mannheim, Germany) using blood samples collected in EDTA tubes and centrifuged for 5 minutes at 3 000 rpm.

#### CoV-2-specific T-cell response

It was measured using flow cytometry. Briefly peripheral blood mononuclear cells were stimulated with S-RBD and N peptide pools. Following stimulation, cells were fixed, permeabilized, stained with monoclonal antibodies against CD3, CD4, CD69, and IFN-γ, and analysed by flow cytometry using a BD FACSCanto II with subsequent analysis in FACSDiva software (Becton Dickinson, Franklin Lakes, New Jersey, USA).

All microbiological tests were conducted at the Institute of Microbiology and Immunology, Faculty of Medicine, University of Ljubljana, Slovenia.

### Adverse events

Adverse events were assessed and graded from 1 to 5 according to the Common Terminology Criteria for Adverse Events (CTCAE) of the National Cancer Institute.

### Key nutritional and physical-functional parameters

Nutritional Risk Screening (NRS), Global Leadership Initiative on Malnutrition (GLIM) criteria, body weight (BW), Body Mass Index (BMI), Fat-Free Mass Index (FFMI), phase angle (PA), and handgrip strength (HGS) were monitored in patients and controls at baseline. In patients, followup assessments were conducted at 3-month intervals over a 12-month period.

### Follow up

Assessments were performed at baseline (prior to the first vaccination), 14 days after the second dose, and at 3, 6, 9, and 12 months following the initial vaccination. The third dose was given at month 6 in patients and at month 9 in controls, with an additional evaluation conducted 14 days after the third vaccination.

Investigators surveyed patients, and blood samples were collected during routine outpatient visits. Averse events were monitored via questionnaires and telephone interviews.

### Ethical aspects

This study was conducted in accordance with the ethical principles of the Declaration of Helsinki and the International Council for Harmonisation of Technical Requirements for Pharmaceuticals for Human Use (ICH-GCP) guidelines. The study and its amendment were approved by the National Medical Ethics Committee of the Republic of Slovenia on March 24, 2021, and October 21, 2021 (Approval No. 0120-109/2021/9). Prior to enrolment, all participants received written information and provided informed consent after being fully informed about the study objectives and procedures. The study was conducted in compliance with all applicable national and international regulations, including the General Data Protection Regulation (GDPR). Participation in the study did not alter or interfere with the treatment of malignant disease. Only individuals who voluntarily consented to both vaccination and participation in the study were included.

### Data analysis

We examined the association between B-cell count and time since rituximab using linear regression in patients who were no longer receiving rituximab at baseline (measurement a0).

Vaccine response was evaluated across four groups: healthy controls and cancer patients with baseline B-cell counts of 0, between 0 and 0.1, and ≥ 0.1 × 10^9^/L (the latter representing a normal B-cell count). For each measurement, we estimated the geometric mean anti-S antibody concentration and its 95% confidence interval. At each time point, anti-S titres were log-transformed to adjust for right-skewness. Means and 95% confidence intervals were calculated on the log scale and then back-transformed, yielding geometric means with their corresponding 95% confidence intervals.

For formal comparisons, we focused on the control group and cancer patients with B-cell counts ≥ 0.1 × 10^9^/L; the remaining B-cell groups were not analysed because they contained too few participants for separate analysis. Linear mixed-effects models were used to assess whether vaccine response differed between these two groups over time. Two nested models were compared to test the group-by-measurement interaction. The first model included group and measurement as fixed effects and participant ID as a random intercept; the second model additionally included the group × measurement interaction. The outcome was log-transformed anti-S, and the models were compared using a likelihood ratio test. In addition, we were particularly interested in whether the two groups differed at time points a1 and b1 (after the first and second vaccination, respectively). Due to the small sample size (only 7 cancer patients were available at a1 and 4 at b1), we used Welch’s t-test on log(anti-S), rather than model-based marginal means, to compare the two groups at a1 and b1.

## Results

Between April 2 and May 28, 2021, a total of 35 patients were initially enrolled into four primary groups:

L1 (n = 5): Rituximab and CHOP regimen, L2 (n = 15): Rituximab maintenance treatment, L3 (n = 5): Rituximab combined with chemotherapy within 24 months prior to the first vaccination and L4 (n = 10): Rituximab monotherapy or in combination with chemotherapy more than 24 months before the first vaccination.

During the study, five patients were excluded for various reasons: one patient from L1 due to protocol violations, two patients (from L2 and L4) lost to follow-up, one patient from L2 who withdrew consent, and one patient from L4 with an extremely low antibody response 10 years after bone marrow transplantation (not a primary exclusion criterion). Due to the limited sample size, the initial four groups were consolidated into two:

Group 1 (on rituximab, n = 17): Patients receiving rituximab during vaccination (4 from L1, 13 from L2) and Group 2 (post-rituximab, n = 13): Patients who had received rituximab previously (5 from L3, 8 from L4).

We excluded measurements from individuals who contracted COVID-19. We also excluded participants with missing anti-S values. Thus, of 30 eligible patients, only 25 were included in the final analysis.

On January 8, 2021, 41 healthy volunteers were enrolled in the control group. Of these, 24 completed the study. The primary reasons for study discontinuation were early administration of the third dose, missing blood samples, and incomplete sample collections.

The patient and volunteer groups were not ideally matched with respect to age and sex (Table 1).

**TABLE 1.** Demographic characteristics of the patient and volunteer groups

Characteristic	Overall N = 66	Patients N = 25	Volunteers N = 41	p-value^1^
Sex: n (%)				0.039
male	17 (26%)	10 (40%)	7 (17%)	
female	49 (74%)	15 (60%)	34 (83%)	
Age: mean (Q1, Q3)	51 (39, 62)	65 (56, 68)	42 (36, 53)	< 0.001

1 Pearson’s Chi-squared test; Wilcoxon rank sum exact test

### Relationship between time since last rituximab and lymphocyte B-cell count

**FIGURE 2.**
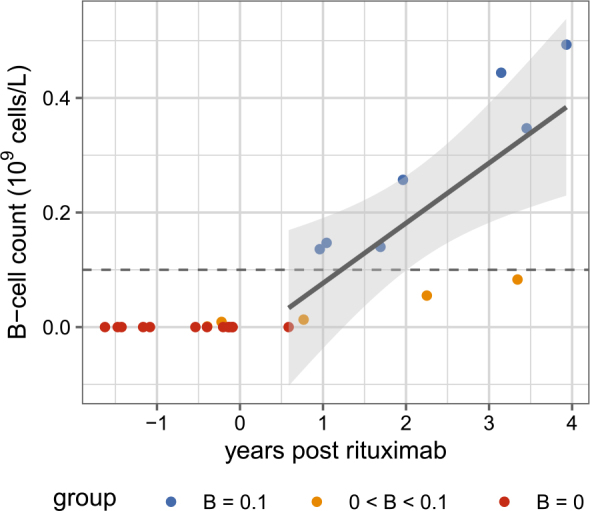
Relationship between time since last rituximab and lymphocyte B-cell count Each point represents a single patient’s measurement. Patients with B-cell counts of 0, 0–0.1, and > 0.1 × 10^9^ cells/L are represented as red, orange, and blue dots, respectively. The solid line shows the fitted linear regression based solely on subjects who have stopped receiving rituximab (i.e., time > 0), and the shaded region indicates the 95% confidence interval for the fitted regression line.

Our first aim was to investigate the relationship between B-cell count and time since the last rituximab administration. Patients were divided into three groups: those with absent B cells, those with B-cell counts below the normal threshold of 0.1 × 10^9^ cells/L, and those with normal B-cell counts. The data are shown in Figure 2. We observe that patients still receiving rituximab (i.e., negative days since rituximab) have a B-cell count of zero. Over time, however, B cell counts gradually increase. This relationship was examined using a linear regression model; the fitted regression line and its 95% confidence interval are shown in Figure 2. Only data from patients who had stopped receiving rituximab (positive days) were analysed. Results indicate that the association between time since rituximab cessation and B-cell count is statistically significant (Table 2), and the adjusted R-squared of the model is 50%.

**TABLE 2.** Linear regression model results of the relationship between B-cell count and time post last rituximab

Term	Estimate	SE	t	df	p	CI lower	CI upper
(Intercept)	-0.03	0.08	-0.37	9	0.718	-0.20	0.14
Years post rituximab	0.10	0.03	3.32	9	0.009	0.03	0.18

CI = confidence interval; SE = standard error

### Relationship between B-cell count and anti-S concentration

**FIGURE 3.**
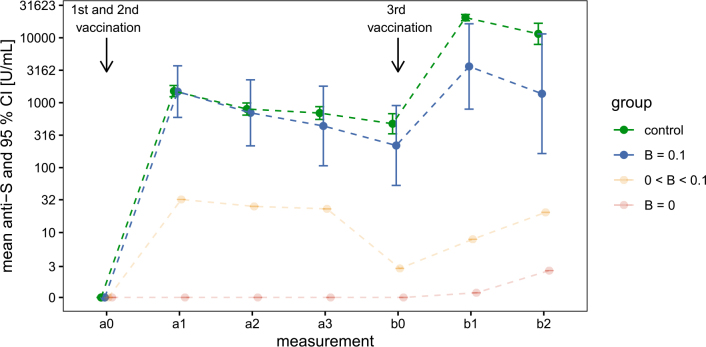
Geometric mean anti-S antibody concentrations in three patient subgroups and healthy volunteers following primary and booster vaccinations Patient subgroups with B-cell counts of 0, 0–0.1, and > 0.1 × 10^9^ cells/L, and healthy volunteers are shown in red, orange, blue, and green, respectively. The mean estimates and their 95% confidence intervals were calculated on the log-transformed scale due to the skewed distribution of the original data, then back-transformed for display. Confidence intervals for the B = 0 and 0 < B < 0.1 groups were omitted because of the limited sample size and the high concentration of zeros.

Our second objective was to evaluate changes in anti-S antibody concentrations following vaccination, and compare these across four groups (patients with B-cell counts of 0, 0–0.1, and > 0.1 × 10^9^ cells/L, and healthy volunteers). Figure 3 presents the geometric mean of antibody concentrations for each time point in each group, along with 95% confidence intervals.

After the first two vaccinations (primary series), the group with B-cell counts > 0.1 displayed a geometric mean response similar to the volunteer group. Although antibody concentration increased after the third vaccination, the rise was significantly lower in patients compared to the volunteer group. Welch’s t-tests, performed on the log-transformed values, showed no statistically significant difference between these two groups at the measurement after completion of the primary vaccination series but revealed statistically significant differences at the measurement following the third vaccination (Table 3). These observations suggest an interaction between measurement time (a1 and b1) and group (volunteers or patients with B-cell counts > 0.1). To formally assess this interaction, we used a mixed-effects model and compared the model with interaction against the model without the interaction. The difference between the two models was statistically significant (χ^2 (1) = 18.96, p < 0.001).

**TABLE 3.** Welch’s t-test results for log-transformed anti-S antibody concentrations (U/mL), comparing the B > 0.1 × 109 cells/L group with the volunteer group at 14 days post-primary and post-booster vaccination

Measurement	Group	Geometric mean (SD)	Welch’s t-test
a1	control	1 510 (650)	t (df = 6.8) = 0.05
	B ≥ 0.1	1 482 (1 079)	p = 0.963
b1	control	20 442 (3 572)	t (df = 3.1) = 3.62
	B ≥ 0.1	3 617 (2 510)	p = 0.035

### CoV-2-specific T-cell response

CoV-2-specific T-cell responses were assessed in patients before and 14 days after both the primary vaccination and the booster dose. However, due to technical issues, all measurements of T-cell interferon responses following stimulation with N and S peptides were unusable.

### Adverse effects

Adverse effects were mostly mild. No significant differences were observed between patient groups and volunteers after the primary and booster vaccination. CTCAE grades after revaccination were significantly higher than those observed after the first dose (mean grade 1,09 *vs*. 0,86, p = 0.011). No significant correlations were found between the anti-S antibody response and the severity of adverse effects at either time point.

### Nutritional and physical-functional parameters

At baseline, anthropometric and functional measurements were compared between cancer patients and healthy volunteers.

Patients had a significantly higher body weight (mean 77.8 ± 16.8 kg) compared to volunteers (mean 69.7 ± 15.0 kg; p = 0.037), as well as a higher BMI (28.0 ± 5.4 *vs*. 24.8 ± 5.1 kg/m^2^; p = 0.012).

Although patients had slightly higher FFMI values (mean 18.8 ± 2.6 *vs*. 17.7 ± 2.4), this difference was not statistically significant (p = 0.306).

A marked difference was observed in phase angle, a parameter reflecting cellular integrity. Patients had significantly lower values compared to controls (mean 5.17 ± 0.88 *vs*. 6.41 ± 0.72; p < 0.0001). The phase angle was slightly lower in the group of patients on rituximab (mean 4.947, SD 0.665) compared to the group of patients after rituximab treatment (mean 5.469, SD 1.051); however, the difference was not statistically significant (p = 0.134) and the sample size was too small to analyse a potential impact of the phase angle on the increase in anti-S antibodies following vaccination.

No significant difference was found in handgrip strength measured by dynamometry (patients: 33.8 ± 9.3 kg, volunteers: 36.3 ± 10.2 kg; p = 0.280), although a trend toward lower strength was observed among patients.

During the 12-month period, there was no clinically significant deterioration or improvement in the nutritional and functional status of the monitored patients.

## Discussion

The number of enrolled patients was very low, so the primary objective was adjusted to allow exploratory analysis. Patients with COVID-19 infection at any time, including during the study, were excluded because their inclusion could have confounded the assessment of vaccine-induced antibody responses. In addition, the small number of infected patients precluded meaningful separate analyses or comparisons with uninfected participants.

Because treatment-naïve lymphoma patients were excluded, extending the recruitment period would likely not have increased the number of patients enrolled in the study.

Due to technical issues, the measurements of CoV-2-specific T-cell responses yielded unreliable results and could not be used.

Adverse effects were generally mild and comparable between cancer patients and healthy volunteers, with slightly higher CTCAE grades observed after revaccination. No association was found between adverse effect severity and the anti-S antibody response.

At baseline, cancer patients had higher body weight and BMI than controls, while no statistically significant differences were observed in FFMI or handgrip strength. In contrast, phase angle was significantly lower in patients, indicating impaired cellular integrity. Due to the limited sample size, more detailed subgroup analyses and assessment of the impact of nutritional and functional parameters on vaccine-induced antibody responses were not feasible.

We observed that patients still receiving rituximab had a B-cell count of zero. However, in patients post rituximab, the number of B cells gradually increased. The association between time since rituximab cessation and B-cell count was statistically significant in our study, consistent with existing evidence(19, 20).

In the subsequent analysis of antibody concentrations following vaccinations, we focused only on patients with normal B-lymphocyte counts because of the small sample size.

After the first two vaccinations (primary series), the group of patients with normal B-cell counts showed a geometric mean response similar to the control group. However, after the third vaccination, the increase in their antibody concentration was substantially lower.

Volunteers had a longer interval between the second and third vaccination than patients, which would be expected to reduce, rather than increase, the observed group difference. Consequently, identical vaccination schedules would likely have resulted in an even larger difference.

Our principal finding is that B-cells, even after their counts normalize post-rituximab therapy, remain functionally inferior to those in a healthy, rituximab-naïve cohort. This defect is not apparent in the modest antibody response to the primary vaccination series but becomes evident in their inability to generate high antibody concentration upon revaccination.

Rituximab, a B-cell-depleting therapy, significantly impacts the humoral immune response to COVID-19 vaccines. However, once B lymphocytes repopulate, patients can develop antibodies in response to COVID-19 vaccination. The literature indicates that the presence of B cells is crucial for seroconversion and antibody production post-vaccination in rituximab-treated patients.^12,13,21^ This response is enhanced when there is a longer interval between the last rituximab dose and vaccination, allowing for B-cell reconstitution.^10,22^ Studies consistently show that the presence of detectable B cells is a strong predictor of a positive serologic response to COVID-19 vaccines in rituximab-treated patients and a minimum of 10 B-cells∕μL (0.01 × 10^9^ cells/L) in peripheral circulation is often required for seroconversion.^12,13^ The interval between the last rituximab treatment and vaccination is critical. A longer duration allows for B-cell reconstitution, which is associated with higher rates of seroconversion.^23-25^ For instance, a period greater than six months from the last rituximab dose to the booster vaccine significantly increases the likelihood of a positive antibody response^26^. Revaccination with additional doses has been shown to improve antibody concentrations more effectively than a single booster dose, especially in patients with detectable B cells.^17,27,28^

Even without a robust humoral response, T-cell-mediated immunity can be induced in rituximab-treated patients. This cellular immunity may provide some degree of protection against COVID-19, highlighting the importance of vaccination even when B-cell numbers are low.^29,31^

In the available literature on oncological patients treated with rituximab, B-cell repopulation has generally been associated with improved vaccine responsiveness; however, the extent and durability of humoral immune recovery following normalization of peripheral B-cell counts, particularly in the setting of booster vaccination, remain insufficiently characterized.

### Clinical implications

Our findings suggest that normalization of peripheral B-cell counts after rituximab should not be used as the sole criterion for assessing vaccine-induced protection in patients with B-cell lymphoma. Despite apparent immune reconstitution, these patients may remain insufficiently protected following booster vaccination. This underscores the need for individualized vaccination strategies, including optimized timing and booster dosing based on immune recovery.

### Strengths

A key strength of this study is the focused assessment of COVID-19 vaccine–induced humoral immunity in patients with B-cell lymphoma who had achieved peripheral B-cell repopulation after rituximab therapy. This design allowed us to demonstrate preserved antibody responses after primary two-dose vaccination, comparable to healthy controls, while identifying a significantly reduced response following booster vaccination. These findings provide clinically relevant evidence that apparent B-cell recovery does not ensure sustained vaccine responsiveness. The prospective design and systematic follow-up, including monitoring of nutritional status, further strengthen the validity of the results.

### Limitations

The primary limitation of this study is the small sample size, which restricts statistical power and broader generalizability. However, given the rarity of the disease and the strict inclusion criteria in a single-center setting, recruitment of a larger cohort was not feasible. In addition, T-cell–mediated immune response data were not interpretable due to technical issues.

### Future directions

Future multicenter studies with larger cohorts are needed to better characterize qualitative aspects of B-cell reconstitution after anti-CD20 therapy. In particular, analyses of B-cell subpopulations and memory responses may help clarify the mechanisms underlying impaired booster responses and support the development of optimized vaccination strategies.

## Conclusions

In patients with B-cell lymphoma treated with rituximab, recovery of peripheral B-cell counts does not ensure sustained humoral immunity after COVID-19 vaccination. Although antibody responses after the primary two-dose vaccination were comparable to those of healthy controls, this response was not maintained after booster vaccination, with significantly inferior antibody levels observed after the third dose. These findings indicate prolonged functional impairment of vaccine-induced immunity beyond numerical B-cell repopulation and underscore the need for individualized vaccination strategies.
